# From virtual attachments to real-world fertility desires: emotional pathways in game character attachment and parasocial relationships

**DOI:** 10.3389/fpsyg.2026.1743080

**Published:** 2026-02-13

**Authors:** Yuan Qi, Gao Jie, Du Yun, Ding Yi Zhuo

**Affiliations:** 1 Anhui Normal University, Wuhu, China; 2 Nanjing University, Nanjing, China

**Keywords:** attachment theory, character attachment, fertility desire, game concentration, parasocial relationships

## Abstract

**Introduction:**

Low fertility has become a global challenge that threatens sustainable social development. In China, fertility intentions among people of marriageable and childbearing age (18–35 years) remain persistently low. A prevailing “risk consciousness” in online culture, together with the tendency for digital activities to substitute for real-world interactions, further weakens young people’s intrinsic motivation to have children. The relationship between simulated gaming environments and real-world fertility desire, therefore, deserves more rigorous investigation.

**Methods:**

Drawing on parasocial relationship theory and attachment theory, we collected questionnaire data from 612 game players. We tested the theoretical model using Partial Least Squares Structural Equation Modeling (PLS-SEM). Measurement properties were examined via confirmatory factor analysis, and direct and indirect effects were evaluated using bootstrapped mediation tests.

**Results:**

Eleven of the sixteen hypotheses were supported. Game Concentration (GC) positively predicted Identification Friendship (IF), Parasocial Cognition (PC), and Parasocial Emotions (PE), but showed no direct effect on Fertility Desire (FD). Mediation analyses revealed significant indirect effects through the affective pathway. The cognitive path involving PC did not yield substantial indirect effects.

**Discussion/conclusion:**

Our findings indicate that simulated-game experiences are linked to FD mainly via affective rather than cognitive pathways. We propose an “Emotional Compensation Hypothesis,” whereby virtual emotional bonds buffer real-world stressors and, in turn, shape reproductive attitudes. This framing positions emotional bonding as a key theoretical lens for exploring the association between virtual gaming experiences and affective orientations toward parenthood, offering preliminary insights into digital-era reproductive attitudes. Future studies should use longitudinal designs and assess the model’s generalizability across diverse contexts.

## 1 Introduction

The long-term equilibrium of population structure and the sustainability of socioeconomic development are strongly correlated with societal fertility levels (John, 2024). However, low fertility has emerged as a global challenge that profoundly affects the prospects of many countries (Fauser et al., 2024). Within this global trend, China’s fertility transition is particularly noteworthy. Recent surveys show that fertility intentions among individuals aged 18 to 35 in China remain persistently low. This trend has raised considerable concern among scholars and policymakers (Qiao et al., 2024). Some researchers argue that online culture exacerbates young people’s anxieties about the economic costs of childbearing, such as housing and education, and about value shifts, including conflicts between individual development and maternal roles. These concerns have become embedded in public discourse as a form of “Risk Consciousness” that suppresses fertility intentions (Li and Zhou, 2025). Furthermore, the substitutive effects of internet-based social and entertainment activities, along with the theatrical dissemination of anxieties related to marriage and childbirth, not only displace time and motivation for real-world courtship and family formation but also foster a pessimistic social atmosphere that further undermines intrinsic drivers of fertility (He et al., 2024).

The role of simulation-based games could be a significant factor in examinations of the complex determinants of Fertility Desire (FD). These games, which realistically depict life-course trajectories, family roles, and caregiving responsibilities (Gates, 2024), have gained considerable popularity among young players in China (Sun, 2024). Many of these individuals are at a pivotal stage in their decision-making regarding marriage and childbearing. Consequently, researchers have utilized simulation games as a tool to investigate the marital and fertility-related choices of young people (Xiang et al., 2023). For instance, Martins (2025) developed the serious game “FACTS,” which incorporates quiz-based interactions within simulated everyday scenarios. This approach aims to enhance adolescents’ understanding of critical issues such as conception probabilities and the risks of infertility (Martins, 2025). Additionally, empirical studies have shown that gamification can effectively support medical students’ learning on topics related to abortion and infertility (Neubacher et al., 2024).

These studies demonstrate the effectiveness of simulation-based games in knowledge transmission and short-term cognitive change. However, their primary aim is to enhance “Reproductive Health Literacy” rather than to influence FD directly. A systematic review by Dinh et al. (2024) that includes 32 studies underscores this limitation by examining infant simulators, which are often used in adolescent pregnancy prevention programs. Although these simulators may temporarily increase awareness of childcare responsibilities, there is insufficient long-term follow-up evidence to support their effectiveness in reducing teenage pregnancy rates or in altering the timing of fertility planning (Dinh et al., 2024). This finding highlights a significant gap: even when simulated experiences produce short-term cognitive effects, they do not necessarily lead to stable, long-term behavioral intentions regarding FD.

Research on simulation games spans a spectrum from educational games, which primarily emphasize knowledge transmission and exhibit relatively straightforward effects, to experiential simulations, which aim to alter attitudes and behaviors but yield more complex outcomes and lack robust long-term evidence (Arima, 2025). Existing studies have predominantly focused on the former category. In contrast, direct and systematic investigations into how simulation games are associated with individuals’ affective orientations toward parenthood—through virtual emotional and cognitive pathways—are notably limited. In light of this gap, the present study aims to explore whether such games relate to fertility-related affective dispositions and to test a hypothesized associative pathway linking virtual experiences to real-world attitudes.

This study adopts an exploratory approach to develop and test a preliminary theoretical model linking simulation game experiences to players’ FD through virtual emotional and cognitive pathways. Central to the proposed framework is a sequential psychological pathway: Game Concentration (GC) → Identification Friendship (IF) → Parasocial Relationships (PSRS). Initially, deep game concentration fosters players’ ability to project their self-concept onto virtual characters, thereby establishing the groundwork for the development of profound relational bonds (Weibel and Wissmath, 2024). This heightened concentration directly promotes the emergence of IF, defined as an emotionally charged connection between players and characters. This connection is characterized by trust and perceived support, resembling real-life friendships and representing a crucial dimension of character attachment (Lewis et al., 2008). Building on this foundation, players may further cultivate more enduring and structured PSRS. These relationships involve one-sided yet socially and emotionally significant cognitive and affective investments in virtual characters that simulate patterns of genuine interpersonal interaction (Costea et al., 2023). This virtual relational chain may significantly activate and reinforce humans’ innate caregiving motivation. From an evolutionary psychological perspective, the care of offspring represents a fundamental adaptive drive (Khushvaktovna and Fayzievna, 2023). This drive is supported by neural reward systems that can be automatically activated by specific infant cues, such as the baby schema (Glocker et al., 2009).

However, simulation games may also diminish caregiving motivation by substituting for real-world needs. In high-pressure social environments, individuals often face substantial economic and social constraints on their ability to engage in actual childbearing. Under these circumstances, the low-cost and highly controllable virtual caregiving experiences and emotional bonds offered by simulation games (Zhang et al., 2025) may serve as a form of psychological compensation. This compensation can partially fulfill emotional needs for intimacy or caregiving-related achievement (Yin and Shen, 2023). While such substitutive gratification may alleviate anxiety stemming from real-life constraints (Du et al., 2017), it may also, at least in the short term, reduce the perceived urgency of translating FD into actual reproductive behavior.

When players engage deeply in virtual caregiving activities, they progress through the “GC → IF → PSRS” pathway. This pathway constitutes a fundamental psychological conduit through which all subsequent effects on real-world motivation are mediated. Within this framework, the caregiving cues and emotional interactions embedded in game content can serve a dual function: they may activate the player’s intrinsic caregiving motivation. Conversely, they may dampen FD by substituting for unmet real-world needs. This dual-function perspective aligns with the established recognition that simulation games can exert either positive or negative effects on real-world motivation, thereby bridging the core psychological mechanism with its divergent outcomes.

The present study aims to examine the interactions among GC, IF, and PSRS and their effects on FD within the context of simulation games. Specifically, the objectives are to: (1) analyze the relationships among GC, IF, and PSRS; (2) investigate the mediating roles among GC, IF, and PSRS, including Parasocial Cognition (PC) and Parasocial Emotions (PE); and (3) provide empirical evidence to inform sociological theory and government fertility-related policy strategies.

## 2 Theoretical framework and hypothesis development

### 2.1 Theoretical framework

#### 2.1.1 Life simulation games

Life simulation games enable players to replicate essential life-course trajectories, including social interactions, romantic relationships, and child-rearing, through character control and a series of ongoing decisions. This format provides a digital experimental space for investigating complex life choices (Feng, 2024). To further elucidate the moderating role of cultural context, this study selects players of Chinese Parents as a representative sample. Chinese Parents is a highly standardized life simulation game, with its core design focused on the systematization and universalization of real-life rules (Gates, 2024). Additionally, it serves as a culturally embedded simulation game, as its mechanics are profoundly correlated with the sociocultural norms, values, and institutional arrangements specific to Chinese society (Schneider, 2024).

Key variables in simulation games have evolved from mere behavioral imitation to include the psychological relationships players form with in-game characters (Gates, 2024). These variables can be categorized into two main types: psychological mechanisms, such as Character Attachment (CA) and PSRS (Hua and Xiao, 2023), and game system design elements, such as caregiving mechanics and narrative strategies (de Lima et al., 2023). Placing these variables within China’s distinctive fertility-desire context shows that their interplay is culturally specific, rooted in traditional notions such as filial piety and intergenerational expectations (Cai and Xie, 2025). Analyzing this layered contextual specificity helps to build a situated framework for understanding the psychological effects of simulation games.

Players of Chinese Parents are selected as the focal research population because of the game’s deep integration of China-specific social rules and pressures. These include educational competition, intergenerational obligations, and the economic costs associated with marriage and childbearing, which contribute to a high degree of cultural embeddedness in its content (Tan and Austin, 2024). Consequently, the game serves not only as an entertainment product but also as a reflective medium that mirrors real-world social tensions and structural constraints (Schneider, 2024). Chinese young adults frequently experience a significant decline in real-world FD, driven by the substantial economic and social costs of childbearing (Li et al., 2024). However, they may simultaneously display strong enthusiasm for virtual caregiving experiences in simulation games such as Chinese Parents (Tan and Austin, 2024). This notable contrast between “Low Real-World FD” and “High Engagement in Virtual Childrearing” makes the game an especially suitable and theoretically rich case for examining how virtual experiences interact with complex social realities and potentially correlate with FD.

#### 2.1.2 Game concentration (GC)

GC is rooted in Flow Theory, initially proposed by Csikszentmihalyi (1975). This theory describes a psychological state of complete immersion in an activity, characterized by intense attentional focus, a distorted sense of time, and heightened intrinsic motivation (Yu et al., 2023). Within the framework of character identification theory, this concept is integrated as a process of cognitive–affective fusion. In this process, players synchronize their sense of self with the game characters through sustained concentration, leading to a shift from “Observing the Character” to “Being the Character.” Cohen (2018) defines character identification as the temporary adoption of a media character’s perspective, goals, and emotions by the audience. GC, as a subdimension of this theory (Cohen, 2018), highlights how immersion intensifies identification. For example, in video game contexts, increased concentration allows players to disregard external distractions, thereby strengthening their empathy and embodied involvement with the character (Guo et al., 2024).

Klimmt et al. (2013) expand this framework by positing that GC serves as a crucial antecedent of “True Identification.” This concept facilitates a shift from passive media consumption to active participation by altering players’ self-perception (Klimmt et al., 2013). In narrative-driven games, elevated levels of GC enhance character embodiment and have been shown to positively affect psychological well-being and promote behavioral change (Slater, 2021). Furthermore, the dimension of GC often intersects with PSRS theory. It is utilized to explore how games simulate real-world social interactions, thereby creating a developmental pathway from concentration to attachment (Dibble et al., 2016).

In the present study, the GC dimension is derived from the PAIS scale (Li et al., 2013). This selection is based on the premise that GC reflects players’ level of immersion during gameplay. Such engagement may enhance a sense of responsibility toward virtual characters, akin to the “Secure Base” concept in attachment theory (Rain and Mar, 2021). To prevent model redundancy and focus on essential mechanisms, GC is considered a subdimension of character identification. In this capacity, GC serves as a vital link between cognitive immersion and emotional embodiment (Ting and Min, 2025). It plays a crucial role in the process by which virtual experiences may influence real-world attitudes and behaviors (Dang, 2023).

#### 2.1.3 Identification friendship

IF is rooted in attachment theory, which initially described the emotional bond between infants and caregivers. When applied to media contexts, this theory highlights how an individual’s attachment to a character can fulfill real social needs (Bowlby, 1969). Lewis et al. (2008) IF as a multidimensional construct of character attachment, encompassing both cognitive dimensions, such as perceived similarity and reciprocity, and emotional dimensions, including trust and a sense of support. Players incorporate characters into their self-social networks through a “Friendship” framework (Lewis et al., 2008). Rain and Mar (2021) expand this concept by integrating adult attachment styles, positing that individuals with anxious attachment are more likely to develop dependencies on characters, akin to the emotional support experienced in real-life friendships. This dimension is distinct from general attachment as it emphasizes “Horizontal” relationships, such as equal friendships, rather than “Vertical” attachments, like those between parents and children. This focus facilitates social simulation and behavioral mimicry within the game (Rain and Mar, 2021).

Lewis et al. (2008) developed a character attachment scale that incorporates IF as a fundamental dimension to elucidate how players establish “Friend” connections in role-playing games (Lewis et al., 2008). Recently, the rise of virtual reality (VR) has prompted studies to highlight the significance of IF within immersive environments. For instance, in narrative-driven games, this dimension enhances mental health and facilitates behavioral transfer through social simulation (Rain and Mar, 2021). The IF dimension frequently intersects with parasocial relationship theory, providing insights into how games address shortcomings in real-world social interactions and create pathways from friendship to attachment. Martinez et al. (2022) investigated the predictive role of IF in narrative games, revealing that it fosters empathy and encourages a transition from virtual character interactions to real-world social intentions (Martinez et al., 2022).

In this study, we focus on the variable of IF within the context of character attachment. This variable effectively captures the social dimension of character attachment, encompassing both connection and commitment. It aligns directly with the fundamental mechanism of PSRS, wherein social cognition provides a cognitive foundation—such as understanding and similarity—while social emotions contribute emotional depth, including empathy and support (Lewis et al., 2008). This selection facilitates hypothesis testing and mitigates variable inflation by suspending doubts and controlling for narrative or manipulative biases that exhibit lower correlation with the social dimension. Empirically, this sub-dimension is widely used in cross-disciplinary research, as it enhances model specificity (Mula-Márquez et al., 2024).

#### 2.1.4 Parasocial relationships

Horton and Wohl (1956) first defined PSRS as one-way emotional connections between audiences and media personalities. This concept differs from Parasocial Interaction (PI), which denotes transient feelings of connection experienced during media exposure (Giles, 2010). Tukachinsky et al. (2020) further conceptualized PSRS as extensions of social relationships rather than substitutes for social deficiencies. They emphasized the association of these relationships with homogeneity, identification, and narrative immersion (Tukachinsky et al., 2020). Additionally, PSRS are linked to attachment theory, with research indicating that attachment styles, such as secure or anxious, influence their formation (Cole and Leets, 1999). The 2025 Cambridge Dictionary online defines “Parasocial” as “relating to or about a person who feels a connection with a celebrity (or characters in books, films, TV shows, or artificial intelligence) whom they do not actually know.” In September 2025, this definition was updated to explicitly include artificial intelligence, reflecting the emergence of a phenomenon that redefines fan and celebrity cultures. With the assistance of AI, the nature of online interactions has evolved, leading to an increase in one-sided, sometimes unhealthy, and intense relationships (Hollanek and Sobey, 2025). The PSRS examined in this study refers specifically to relationships between players and non-AI-driven, pre-scripted narrative virtual characters.

While the study of PSRS originated within broadcast media, scholars have extended this line of inquiry to life simulation games (Yapp and Tan, 2025). Central to this phenomenon is the ability of simulation games to foster high immersion through role-playing, sustained emotional engagement, and the simulation of social interactions (Liu, 2024). These elements systematically construct virtual environments that fulfill the essential criteria for the formation of PSRS. Consequently, the PSRS that emerges between players and virtual characters extends beyond the conventional one-way viewing experience typical of media. They evolve into more personalized, interactive, and emotionally rich connections (Martin and Cohen, 2023). Thus, our model prioritizes simulation game-based mechanisms.

The formation of PSRs is shaped by multidimensional antecedents, including individual psychological traits (e.g., loneliness, social anxiety) (Liu, 2024), media usage patterns, and contextual affordances (Mula-Márquez et al., 2024). This theoretical foundation situates our work within a broader scholarly discourse. However, we intentionally narrow the scope of the present study to focus on in-game experiential variables—specifically concentration and identification—as proximal, manipulable determinants independent of stable user characteristics. Although PSRs can yield both beneficial outcomes (e.g., social compensation) and potential risks (e.g., reality dislocation) (Tukachinsky et al., 2020), the current framework treats PSRs primarily as adaptive social surrogates within a simulated caregiving context; considerations of maladaptive consequences lie beyond the immediate research question.

#### 2.1.5 Fertility desire

FD is a complex decision correlated with the interplay of macro social contexts, micro individual factors, and increasingly significant digital psychological experiences (Spéder and Bálint, 2024). The introduction of parasocial relationship theory into fertility studies does not diminish the relevance of traditional economic or sociological variables, such as education, career, and family support (Cai and Xie, 2025). Instead, it highlights that, in a highly mediated society, an individual’s psychological reality and perceptions of relationships are crucial variables that affect their real-life planning (Firth et al., 2024). Consequently, this study incorporates PSRS as a variable in simulation games to examine its impact on players’ FDs.

The PSRS scale typically comprises three dimensions: PE, which denotes emotional investment in media or virtual characters (for example, affection or empathy); PC, which involves cognitive processing such as understanding a character’s motivations or recognizing similarities; and PI, which refers to perceived one-way interaction—for instance, the sense that a character is “responding” (Horton and Wohl, 1956). Because PI emphasizes immediate perceived experiences, whereas FD reflects long-term behavioral intentions tied to deeper cognitive and emotional factors (Robertson et al., 2023), we concentrate on the two PSRS dimensions most relevant to FD: PC and PE. Furthermore, GC and IF are conceptualized as immediate psychological responses elicited by the in-game context. Their primary theoretical role is to mediate the effects of game design features (e.g., narrative structures, character interactions) on players’ subsequent attitudinal outcomes, such as FD.

This study develops and evaluates an integrated theoretical framework that proposes psychological pathways through which engagement with simulation games may relate to players’ fertility desires. The framework hypothesizes a mediating factor that links experiences in virtual environments to affective components of real-world fertility attitudes.

First, Simulation games, as a distinct media environment, offer a highly interactive and narrative-rich context that facilitates role-playing and relationship simulation (Feng, 2024). Within this framework, the player’s GC, defined as a state of deep immersion or flow, serves as a crucial psychological catalyst (Yu et al., 2023). This enhanced cognitive and emotional engagement prompts players to project their self-concept onto the virtual character (Gates, 2024). Consequently, this projection intensifies their IF with the character, characterized by trust, support, and perceived similarity, thereby mirroring the dynamics of real-life friendships (Rain and Mar, 2021).

Second, the high-quality IF constitutes the emotional core of PSRS between players and virtual characters (Kilpatrick et al., 2023). In this study, PSR is defined as an ongoing one-way emotional engagement, referred to as PE, and a cognitive understanding of the character, termed PC (Tukachinsky et al., 2020). This relation is hypothesized to bridge the gap between virtual experiences and real-world intentions, potentially influencing players’ long-term attitudes and behavioral intentions toward child rearing, specifically their FD.

Consequently, this theoretical framework delineates a clear mediating pathway: “Simulation Game (media environment) → GC (immersion) → IF (emotional bond) → PSRS (relational mechanism) → FD (real-world intention).”

### 2.2 Hypothesis development

In the realm of virtual gaming, high GC blurs the distinction between reality and the virtual environment, enabling players to experience a profound sense of presence through role immersion. This immersion can facilitate the development of PSRS, as players gain insights into characters’ motivations and emotional responses. Consequently, this understanding may influence adjustments in real-world intentions, such as shifting from virtual social motivations to actual interpersonal interactions (Martinez et al., 2022). This transfer mechanism underscores the role of GC in linking the virtual and the real, thereby driving changes in behavioral intentions through immersive processing (Mula-Márquez et al., 2024). Recent research further corroborates that GC enhances Parasocial Involvement in interactive settings, positively impacting behavioral transfer by cultivating enduring effects on one-sided connections through immersion (Chang et al., 2024). An alternative perspective posits that GC stimulates intrinsic motivations within games, extending the virtual experience into the real world and thereby strengthening intention formation (Martinez et al., 2022). In light of these findings, we propose the following hypotheses:

*H1*: GC positively contributes to PC.

*H2*: GC positively contributes to PE.

GC, a fundamental aspect of embodiment identification, signifies players’ immersive engagement and focused attention within the virtual environment. This mechanism fosters a perception of virtual characters as social entities akin to friends through repeated interactions and narrative immersion, thereby establishing perceived social connections (Breien and Wasson, 2021). Specifically, elevated levels of GC may diminish the boundary between reality and virtuality, enhancing character similarity and resonance, which in turn reinforces IF. This hypothesis is supported by research on narrative-driven games, which indicates that immersive experiences enhance perceptions of relationships. For instance, in role-playing games, players cultivate enduring social bonds through profound involvement (İskender, 2023). Therefore, we propose the following hypothesis:

*H3*: GC positively contributes to IF.

This study posits that GC positively correlates with players’ FD. The primary mechanism underlying this relationship is that GC fosters a profound sense of presence (Blackman, 2024). This presence blurs the distinction between reality and the virtual environment, allowing players to immerse themselves deeply in their characters (van Brakel et al., 2023). Such a strong experience of presence in the virtual realm provides a vital psychological foundation for transferring game-based experiences into real-world decisions. This transfer may alter behavioral intentions, as players derive decision-making motivations from virtual simulations (Jans et al., 2023). Ultimately, the psychological fusion and transfer process driven by GC can effectively mediate and enhance players’ FD (Martinez et al., 2022). Therefore, we propose the following hypothesis:

*H4*: GC positively contributes to FD.

By fostering players’ perceived similarity and emotional support toward virtual characters, the intensity and depth of one-way connections are significantly enhanced. This mechanism is theoretically grounded in the media extension of attachment theory. Here, IF denotes players’ perception of the character as a “Friend,” leading to a reciprocal illusion and emotional attachment (Milman and Mills, 2023). In the gaming context, elevated levels of IF blur the boundaries between virtual and authentic experiences. Players engage in PC, which involves understanding the character’s motivations, and in PE, which encompasses empathy and support, through role immersion. This engagement positively influences PSRS and establishes a pathway from virtual involvement to real-world decision-making (Mula-Márquez et al., 2024). This mechanism underscores IF as a critical bridge that enhances the durability of PSRS, particularly in narrative-driven games, thereby increasing player loyalty and imitation of player behavior (Zhou, 2021). Recent studies further confirm that IF is a positive predictor of PE in virtual environments, helping address real-world social gaps and strengthen relationships (Hoffner and Bond, 2022). Therefore, we propose the following hypotheses:

*H5*: IF positively contributes to PC.

*H6*: IF positively contributes to PE.

IF refers to the process by which players perceive virtual characters as intimate partners, establishing one-sided emotional connections characterized by trust and support. This process fulfills social needs and encourages translating intentions into real-world actions (Rain and Mar, 2021). Within the context of simulation games, high levels of IF foster deep role interactions, creating a framework defined by PE. This framework effectively diminishes the perceived divide between the virtual and real worlds (Chang et al., 2024). Martinez et al. (2022) demonstrated that in life simulation games, such as Animal Crossing, interactions centered on shared goals enhance emotional bonds among players, thereby positively impacting real-world family relationships. When players cultivate strong IF with virtual caregiving characters, they may bridge real-world social gaps through relationship simulation. This process can lead to more positive expectations and intrinsic needs regarding real-world parent–child relationships (Martinez et al., 2022). Consequently, IF may foster stronger PE in virtual environments, which, according to theoretical perspectives on emotional transfer, could be associated with more positive affective orientations toward parenthood. Based on this reasoning, we propose the following hypothesis:

*H7*: IF positively contributes to FD.

PE, including empathy and perceived support derived from interactions with game characters, is positively associated with FD, which we conceptualize as an affective orientation toward parenthood. These emotions positively enhance FD by simulating relationships. This emotional pathway encompasses both emotional fulfillment and motivational transfer (Brehm and Schneider, 2019), which may increase the attractiveness of real-world childbearing in virtual parenting contexts. Existing models underscore the importance of emotional factors in shaping fertility intentions (Bachrach and Morgan, 2013). Consequently, we propose the following hypotheses:

*H8*: PC positively contributes to FD.

*H9*: PE positively contributes to FD.

In narrative-driven games, players’ deep immersion, referred to as GC, initially enhances their IF with virtual characters (Milman and Mills, 2023). This IF, defined by interdependence and support, acts as a crucial link between virtual engagement and more profound social psychological processes (Breien and Wasson, 2021). Furthermore, high-quality IF within games is a significant predictor of players’ social capital and sense of belonging (Choi et al., 2025). Similarly, cooperative games improve the quality of friendships through positive interactions (Pham et al., 2023). Collectively, these findings support a coherent argument: GC may affect players’ PSRS by fostering IF with virtual characters. Therefore, we propose the following hypotheses:

*H10*: IF mediates the relationship between GC and PC.

*H11*: IF mediates the relationship between GC and PE.

Utilizing the framework of embodiment identification, character attachment, and PSRS, we investigate the mediating role of PSR dimensions—specifically social cognition and social emotions—in the relationship between GC and FD. GC serves as a precursor that enhances real-world intentions through the mediating mechanisms of PC, which involves intent modeling, and PE, characterized by empathy support (del Valle-Canencia et al., 2022). Players’ focused interactions and self-identification within games facilitate the establishment of positive virtual relationships, which in turn reshape cognition and influence real-world beliefs (Kackar, 2024). This pathway is particularly pronounced in educational simulation games, where cognitive processing via virtual character relationships significantly enhances behavioral intentions (Tukachinsky et al., 2020). Furthermore, this mediation encompasses mechanisms of emotional fulfillment, as players engage in virtual relationships that evoke emotional connections (Dirin and Laine, 2023), such as those experienced through virtual parenting. Consequently, we propose the following hypotheses:

*H12*: PC mediates the relationship between GC and FD.

*H13*: PE mediates the relationship between GC and FD.

IF may mediate the relationship between GC and FD. This mediation occurs primarily by enhancing players’ perceived reciprocity and emotional closeness toward virtual characters. Consequently, this process facilitates the transfer of immersive gaming experiences into real-world behavioral intentions. This theoretical framework is grounded in the application of attachment theory to digital media. High levels of GC diminish the sense of virtual separation. Through IF, players cultivate a PE framework, which mediates the transition to FD. This reflects the continuity from virtual relationships to real-world decision-making (Milman and Mills, 2023). This mechanism positions IF as a catalyst that reinforces PSRS stability, particularly in simulation games. It strengthens players’ imitation of family roles and internalizes their motivations. Recent research has demonstrated that IF mediates the relationship between GC and PE on virtual platforms. This mediation fills real-world social gaps through relationship simulation and enhances behavioral motivations, such as fertility intentions (Yu et al., 2023). Based on this evidence, we propose the following hypothesis:

*H14*: IF mediates the relationship between GC and FD.

We investigate the mediating role of dimensions of PSRS, specifically social cognition and social emotions, in the relationship between IF and FD. Through IF, players simulate virtual relationship dynamics, which in turn reshape their real-world cognition (Milman and Mills, 2023). By understanding and empathizing with the experiences of virtual characters—such as parenting motivations, challenges, and joys (Liu, 2024)—players can construct or modify their cognitive schemas, attitudes, and knowledge regarding “Parenthood” (Cockerill et al., 2024). This process, grounded in cognitive commitment, allows players to achieve relationship satisfaction and emotional support through attachment and emotional resonance with virtual characters (Feng, 2024). Such positive emotional experiences may subsequently relate to the concept of “Parenting,” thereby enhancing the emotional desire for childbearing and fostering caregiving behaviors in real life (Zhang et al., 2024). This, in turn, influences real-world behavioral decisions and ultimately affects FD (Zhou, 2021). Therefore, we propose the following hypotheses:

*H15*: PC mediates the relationship between IF and FD.

*H16*: PE mediates the relationship between IF and FD.

These 16 hypotheses collectively delineate a clear trajectory: GC enhances players’ ability to form high-quality relationships with virtual characters, specifically through IF and PSRS. The positive experiences derived from these virtual interactions may subsequently affect players’ macro-level social intentions in the real world, such as FD. Furthermore, the seven mediation hypotheses provide a process-oriented analysis of the previously mentioned direct relationships. Together, these findings support a central premise: GC does not directly alter FD; rather, it correlates with this outcome by fostering specific, high-quality social and emotional connections, namely IF and PSRS, within the gaming environment.

## 3 Research method

### 3.1 Research model

This study integrates Avatar Identification Theory and Parasocial Relationship Theory to propose a preliminary mediation model linking engagement with simulation games to FD through virtual emotional and cognitive pathways. Avatar Identification Theory asserts that deep immersion, characterized by GC, allows players to assimilate their self-concept into virtual characters (Yu et al., 2023). This process facilitates a transition from merely controlling the character to “Becoming” the character, serving as the foundation for establishing a profound connection (Martinez et al., 2022). Expanding on this notion, the IF sub-dimension of Role Attachment Theory delineates this connection, wherein players cultivate a horizontal emotional bond with the character that encompasses trust and support, similar to real-life friendships (Rain and Mar, 2021). Furthermore, PSRS Theory elaborates on this identification by structuring it into sustained, one-sided cognitive and emotional investments. PC pertains to the comprehension of the character’s motivations (Cockerill et al., 2024), while PE encompasses empathy and affection. Both aspects have been demonstrated to potentially influence real-world attitudes and behaviors (Wu et al., 2025).

**Figure 1:**
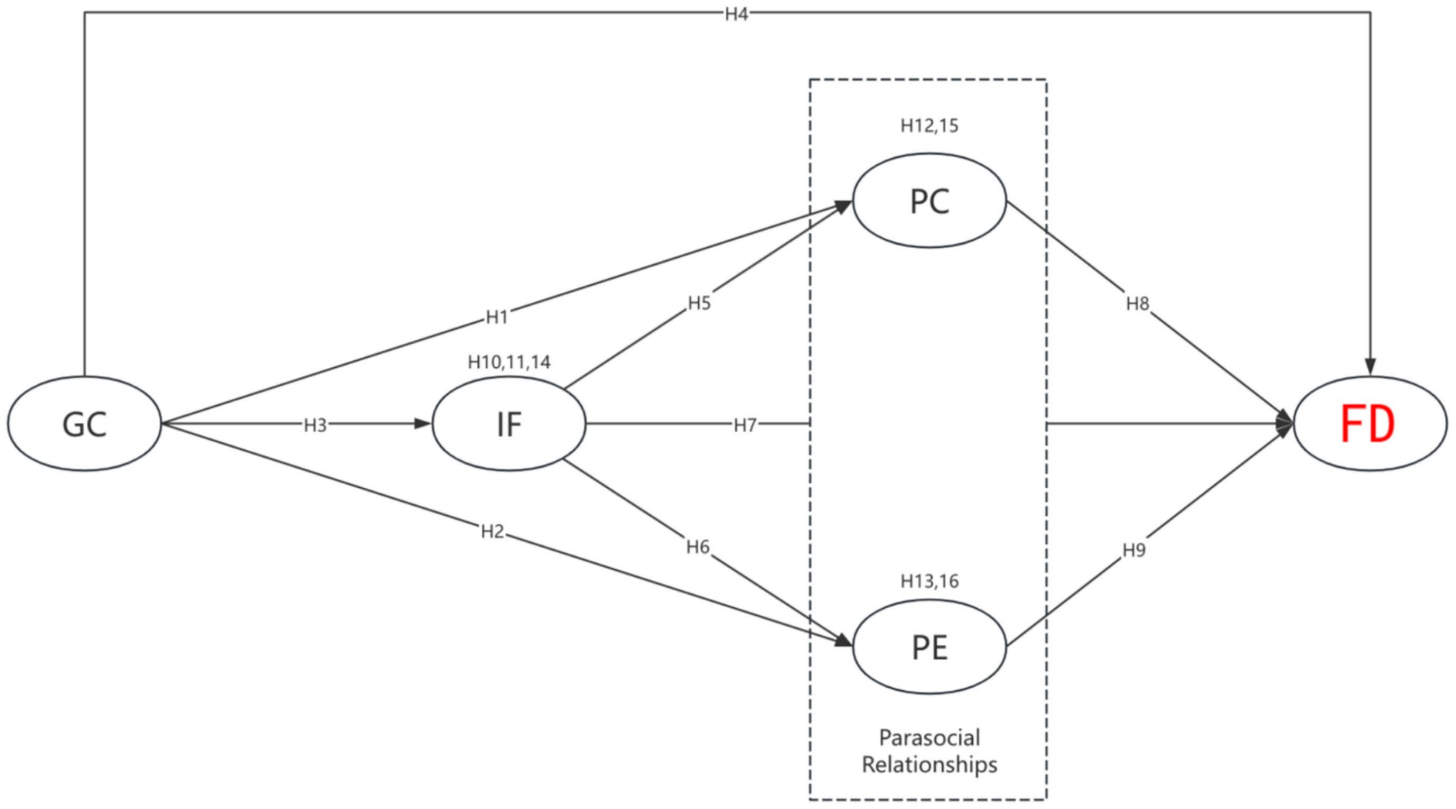
Research model and hypotheses. Game concentration (GC); identification friendship (IF); parasocial cognition (PC); parasocial emotions (PE); fertility desire (FD).

This theoretical framework simultaneously guides and delineates the core model path of this study (See Figure 1). The underlying logic posits that various theories elucidate critical stages in the ongoing transition from virtual immersion to real-world impact. GC, derived from Avatar Identification Theory, elucidates how profound immersion in the game initiates the entire psychological process (Yu et al., 2023). IF, based on Role Attachment Theory, characterizes the emotional bond established between the player and the character (Rain and Mar, 2021). Additionally, PSRS elucidates how this virtual bond affects real-world attitudes and behaviors through cognitive and emotional mechanisms (Wu et al., 2025).

The study proposes the following model: GC positively correlates with IF with virtual characters. This IF subsequently promotes PC and PE toward the character. Ultimately, these two dimensions of PSRS may affect the player’s real-world FD. The model suggests that virtual simulation, serving as a platform for psychological behavioral experiments, may subtly reshape an individual’s cognitive and emotional perspectives on family roles and parenting behaviors. This occurs through the pathway “GC →IF → PSRS → FD” in a low-fertility society.

This study relies on cross-sectional data, which permits only the examination of the correlational structure among variables and does not facilitate the establishment of a definitive causal chain. Consequently, any causal inferences drawn from these conclusions require further validation through alternative research designs.

### 3.2 Sample and data collection

Existing research indicates that video games play a significant role in the lives of adolescents, while fertility attitudes among young adults are increasingly concerning. Consequently, this study focuses on players aged 18 to 35. Data collection commenced in April 2024, with the sample drawn from gaming communities associated with the game “Chinese Parents” on platforms such as BiliBili, Baidu Tieba, QQ, and WeChat, as well as university students from Anhui, Jiangsu, and Sichuan provinces. This approach aimed to encompass a diverse geographic range to mitigate regional bias. Before the formal study, informed consent was obtained from participants, and specific restrictions were implemented, such as limiting participation to “Chinese Parents” players. A preliminary study was conducted to refine the questionnaire by recruiting 100 players for a pilot test. This process resulted in revisions that clarified ambiguous questions, incorporated reverse-coded items, eliminated redundant items, and restructured the logical flow to ensure the survey was both concise and compelling.

We obtained student lists from university academic secretaries and had counselors randomly distribute 1,100 questionnaires via QQ and WeChat. This effort yielded 260 valid responses, resulting in a response rate of 23%. Additionally, we collected 352 responses from gaming community platforms, bringing the total number of valid questionnaires to 612. Regarding demographics, 260 respondents identified as male and 352 as female. Among the participants, 20% were under 20 years old, 41% were aged 20–23, and 39% were aged 23 or older. Detailed demographic data are presented in Table 1.

**Table 1:** Demographic statistics table, containing age, gender, and game duration.

Characteristic	Total Sample (*n* = 612)	Mean (SD) or %
Age (years)		
<20	122	20
20–23	251	41
>23	238	39
Gender (%)		
Male	260	42
Female	352	58
Other	0	0
Usage time		
<One year	302	49
One year–three years	247	40
>Three years	63	11

### 3.3 Measurement

Data were collected through a questionnaire survey of players, and the proposed model was tested using Partial Least Squares Structural Equation Modeling (PLS-SEM) rather than covariance-based SEM. PLS-SEM is a variance-based, nonparametric analytical approach (Kanchanawongpaisan, 2024). Given the exploratory nature of this study, the inclusion of a complex mediation model with multiple latent constructs, and the violation of normality assumptions in the data distribution, PLS-SEM was deemed more suitable for model estimation and hypothesis testing. Data were collected via an online survey and analyzed primarily using PLS-SEM (Smart PLS 4), in accordance with established guidelines for reflective measurement models. SPSS 27 was employed for initial data cleaning and descriptive analysis. The survey questionnaire was adapted from the relevant literature and used a 5-point Likert scale ranging from strongly disagree to agree strongly. Preliminary normality tests indicated that all constructs exhibited slight negative skewness (ranging from −0.976 to −1.406) and moderate kurtosis (ranging from −0.065 to 1.067), and PLS-SEM is more robust to non-normal data.

The independent variables in this study are GC, IF, PC, and PE. The dependent variable is FD. We utilize a questionnaire survey methodology to investigate the relationships among GC, IF, PC, PE, and FD.

IF was measured using an adapted version of the game character attachment scale developed by Lewis et al. (2008), which was subsequently translated and validated by Chinese researchers, including Wei Hua. The scale exhibited strong reliability and validity (Lewis et al., 2008). It comprises six items related explicitly to IF. In this study, the internal consistency coefficient (*α*) was 0.92.

PSRS: PSRS was measured using scales developed by Schramm and Hartmann (2008) and Rubin et al. (1985). The statements and descriptions were modified and refined in accordance with prior research to create the initial scale utilized in this study. The final scale comprises two dimensions: PE and PC, totaling six items (Schramm and Hartmann, 2008). The internal consistency coefficient (α) for this study was 0.80. GC: GC was assessed using the Media Character Identification Scale developed by Cohen (2018), which includes a sub-dimension specifically for GC (Cohen, 2018). This sub-dimension consists of two items, and the internal consistency coefficient (α) was 0.86.

FD was assessed using the FD Scale, which encompasses both Economic Practicality FD and Emotional FD. Chasiotis et al. (2006) demonstrated that affection for children positively predicts fertility intentions among young couples. Within the same cultural framework, emotional value emerges as the most significant factor (Chasiotis et al., 2006). According to the Theory of Planned Behavior (Ajzen, 1991) and recent work on fertility motivation (Yu et al., 2023), an individual’s affective stance toward parenting scenarios is a primary determinant of fertility intention: positive emotional preferences increase the intention to have children, while negative emotions suppress it (McAllister et al., 2016). Consequently, this study modified the FD Scale to emphasize the Emotional FD sub-dimension. This sub-dimension comprises three items, with an internal consistency coefficient (α) of 0.88.

The literature indicates that age, gender, and the duration of game play may be associated with an individual’s FD. Consequently, these factors were treated as control variables in this study (see Table 2).

**Table 2:** Scale items, including items for identification/friendship, PSRS, GC, FD, etc.

Latent variable	Measurement items	References
IF	I sometimes forget my own feelings and take on my character’s.	Lewis et al. (2008)
	I enjoy pretending my character is a real person.	
	I consider my character a friend.	
	I enjoy pretending I am my character.	
	I could see myself being attracted to my character.	
	I daydream about my character.	
PE	I am very worried about what the avatar I have developed will encounter in the next mission.	Schramm and Hartmann (2008)
	There are situations where I wish I could tell the development character what to do.	Ramasubramanian and Kornfield (2012)
	I hate people who hurt avatars in games	
PC	When I play the game, I feel it has something to do with character development.	Schramm and Hartmann (2008)
	When I am not playing games, I always think about this character development.	Ramasubramanian and Kornfield (2012)
	When the character is happy, I am happy too.	
GC	I forgot myself in the game	Cohen (2018)
	In the game, I feel like I am in the game world	Li et al. (2013)
FD	I feel happy watching my children grow up	Chasiotis et al. (2006)
	It is fun to have a kid around you	
	Having a child to love and care for	

## 4 Results

### 4.1 Measurement model

The initial stage involved evaluating the measurement model using SPSS 27 (Sen and Yildirim, 2022). This assessment focused on the reliability, convergent validity, and discriminant validity of the model variables. Table 3 displays the mean, standard deviation, Cronbach’s alpha, Composite Reliability (CR), Average Variance Extracted (AVE), and factor loadings for the items within the conceptual model. Cronbach’s alpha and CR values are commonly employed to evaluate reliability (Hair and Alamer, 2022). According to Fornell and Larcker (1981), the CR value should exceed 0.60, in conjunction with Cronbach’s alpha. The results presented in Table 3 indicate that the Cronbach’s alpha values for the five constructs in the conceptual model ranged from 0.863 to 0.929, while the CR values varied from 0.894 to 0.948. All values surpassed the critical threshold of 0.60, demonstrating high reliability (see Table 4).

**Table 3:** Assessment of the construct measurement.

Latent variable	Items	Loadings	VIF	Cronbach’s α	CR	AVE
GC	GC_1	0.929	2.358	0.863	0.936	0.879
	GC_2	0.946	2.358			
IF	IF_1	0.860	2.86	0.929	0.944	0.738
	IF_2	0.884	3.061			
	IF_3	0.848	2.703			
	IF_4	0.876	2.914			
	IF_5	0.850	2.611			
	IF_6	0.836	2.418			
PC	PC_1	0.899	2.371	0.867	0.918	0.788
	PC_2	0.904	2.239			
	PC_3	0.859	2.172			
PE	PE_1	0.902	2.547	0.875	0.923	0.800
	PE_2	0.902	2.509			
	PE_3	0.879	2.145			
FD	FD_1	0.886	2.496	0.884	0.928	0.810
	FD_2	0.903	2.645			
	FD_3	0.911	2.376			

**Table 4:** Discriminant validity of the measurement model.

Constructs	GC	IF	PC	PE	FD
GC	0.938				
IF	0.168	0.859			
PC	0.136	0.162	0.888		
PE	0.181	0.157	0.118	0.895	
FD	0.095	0.117	0.125	0.173	0.900

#### 4.1.1 Common method bias (CMB) assessment

To address concerns about potential standard-method bias (CMB), a risk associated with single-source self-reported questionnaire data, we used Harman’s single-factor test, a well-established diagnostic method for CMB (Podsakoff et al., 2003). This process involved performing an unrotated exploratory factor analysis (EFA) on all measurement items, which comprised 17 items across five constructs in the present study. The results of this analysis are summarized as follows: the first unrotated common factor accounted for 16.642% of the total variance. This percentage is significantly lower than the 40% threshold often cited as indicative of substantial CMB (Podsakoff et al., 2003; Spector, 2006). Consequently, these findings suggest that CMB does not pose a significant threat to the validity of the observed relationships among constructs in this dataset.

The results of this test indicate that the first unrotated common factor accounted for 16.642% of the total variance. This percentage is significantly lower than the 40% threshold often regarded as indicative of meaningful standard method bias CMB (Podsakoff et al., 2003; Spector, 2006). Consequently, these findings suggest that CMB does not pose a significant threat to the validity of the observed relationships among constructs in this dataset.

### 4.2 Structural equation modeling

Due to multicollinearity in the structural model, PLS-SEM path coefficients may be biased. To address this issue, we examined multicollinearity by assessing the variance inflation factor (VIF) for each measurement item. VIF values should not exceed 5 (Hair and Alamer, 2022). The results show that all VIF values in the model are below this threshold, indicating no multicollinearity.

**Figure 2:**
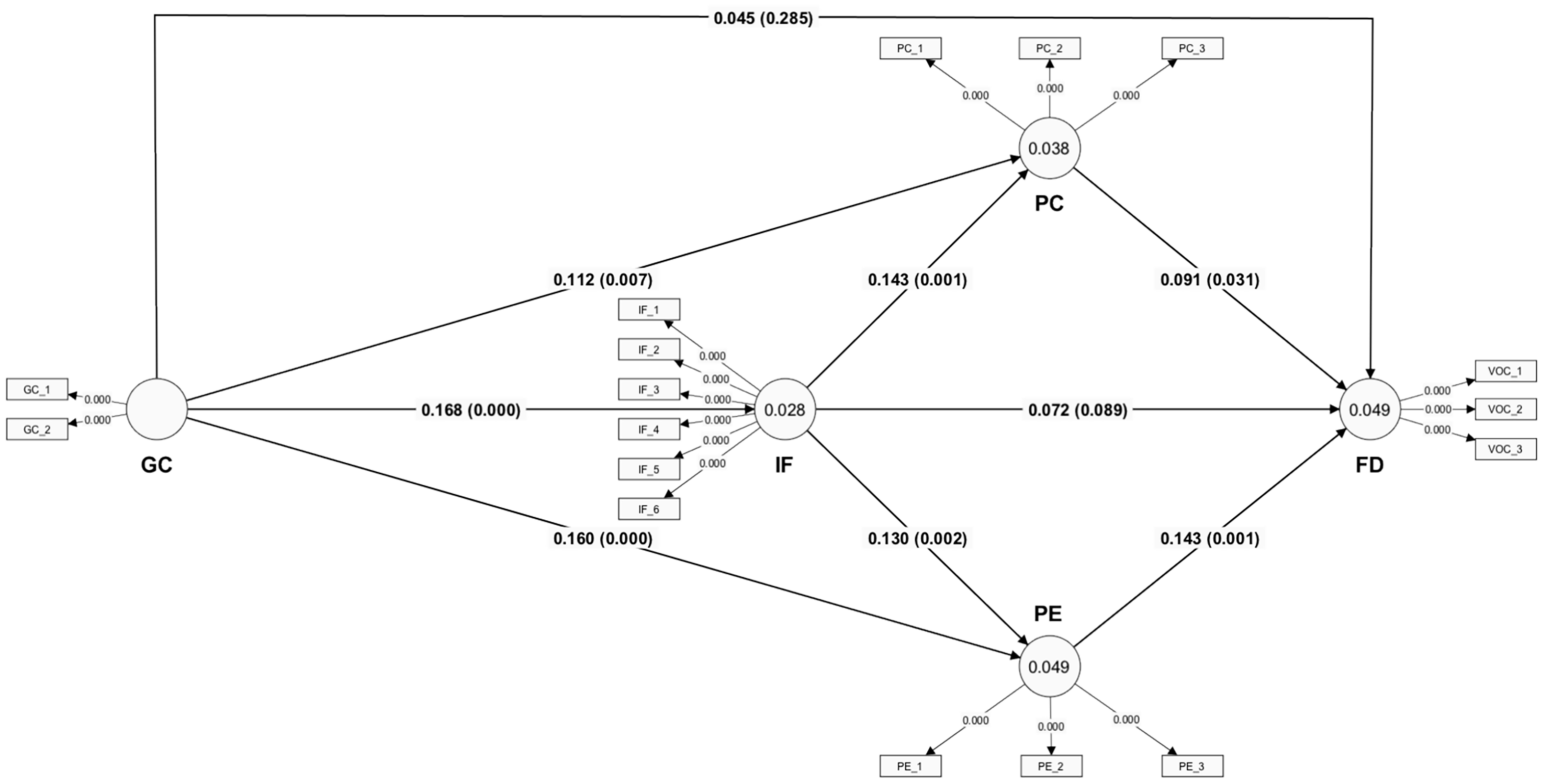
Results of the PLS-SEM analysis.

To examine the path coefficients, their significance levels, and t-values, we employed a bootstrapping method with 5,000 subsamples. The results indicate that GC significantly contributes to PC (H1), PE (H2), and IF (H3), but not FD (H4). IF significantly contributes to PC (H5) and PE (H6), but not FD (H7). PC (H8) and PE (H9) both significantly contribute to FD. For more details, please refer to Figure 2. Table 5 presents the final hypothesis testing results.

**Table 5:** Results of hypothesis testing.

Hypothesis	Structural path	Path coefficient	*T*-values	*p*-values	Confidence interval (95%)		*f*-square	Supported
					2.50%	97.50%		
H1	GC → PC	0.168	2.712	0.007**	0.033	0.195	0.013	YES
H2	GC → PE	0.160	4.122	0.000***	0.084	0.237	0.026	YES
H3	GC → IF	0.168	3.992	0.000***	0.089	0.253	0.029	YES
H4	GC → FD	0.045	1.070	0.285	−0.038	0.126	0.002	NO
H5	IF → PC	0.143	3.372	0.001**	0.062	0.230	0.021	YES
H6	IF → PE	0.130	3.111	0.002**	0.052	0.217	0.017	YES
H7	IF → FD	0.072	1.700	0.089	−0.009	0.156	0.005	NO
H8	PC → FD	0.091	2.154	0.031*	0.008	0.175	0.008	YES
H9	PE → FD	0.143	3.278	0.001**	0.058	0.231	0.020	YES

*p < 0.05; **p < 0.01; ***p < 0.001.

To evaluate the overall fit of the constructed theoretical model, this study employs multiple fit indices. The results show that the standardized root mean square residual (SRMR) between the saturated and estimated models is 0.039 and 0.041, respectively, both below the acceptable threshold of 0.08, indicating good absolute fit (Hu and Bentler, 1999). Additionally, the non-normed fit index (NFI) is 0.878, approaching but slightly below the recommended standard of 0.90, suggesting that the model performs adequately in relative fit aspects. Although the chi-square value (χ^2^ = 823.843) is significant, considering that the chi-square test is susceptible to influence from large sample sizes, its interpretation should be combined with other indices for comprehensive judgment. The d_ULS and d_G values are 0.258 and 0.196, respectively, indicating that the model is robust in parameter estimation.

Further analysis of the explanatory power of endogenous constructs reveals that the *R*^2^ values for IF, PC, PE, and FD are 0.028, 0.038, 0.049, and 0.049, respectively, with adjusted *R*^2^ values of 0.027, 0.036, 0.046, and 0.043. Although the overall explanatory power is low, it still indicates that the antecedent variables in the model have a certain degree of predictive effect on each outcome variable, particularly demonstrating weak but significant explanatory capacity in the aspects of PC and FD.

Additionally, the model’s predictive relevance is evaluated through cross-validated redundancy (*Q*^2^), with results showing: the *Q*^2^ value for GC is 0.000, indicating a lack of external predictive capability; whereas the *Q*^2^ values for IF, PC, PE, and FD reach 0.020, 0.028, 0.037, and 0.035, respectively, all greater than 0, suggesting that these constructs possess a certain degree of external predictive efficacy, supporting the model’s predictive validity (Hair and Alamer, 2022).

The mediating effects proposed in H10–H16 were examined using bootstrapping procedures in PLS-SEM. Table 6 summarizes the direct, indirect, and total effects, along with variance accounted for (VAF) values and mediation types.

**Table 6:** Mediation analysis results.

Hypothesis	Paths	Direct effect (*t*, *p*)	Indirect effect (*t*, *p*)	Total effect (*t*, *p*)	VAF (%)	Interpretation	Results
H10	GC → IF → PC	0.112 (2.712, 0.007)	0.024 (2.379, 0.017)	0.136 (3.292, 0.001)	17.60%	Partial mediation	Significant
H11	GC → IF → PE	0.16 (4.122, 0.000)	0.022 (2.215, 0.027)	0.181 (4.588, 0.000)	12.20%	Partial mediation	Significant
H12	GC → PC → FD	0.045 (1.07, 0.285)	0.01 (1.665, 0.096)	0.095 (2.23, 0.026)	10.50%	No mediation	Not significant
H13	GC → PE → FD	0.045 (1.07, 0.285)	0.023 (2.378, 0.017)	0.095 (2.23, 0.026)	24.20%	Full mediation	Significant
H14	GC → IF → FD	0.045 (1.07, 0.285)	0.012 (1.462, 0.144)	0.095 (2.23, 0.026)	12.60%	No mediation	Not significant
H15	IF → PC → FD	0.072 (1.7, 0.089)	0.013 (1.707, 0.088)	0.104 (2.505, 0.012)	12.50%	No mediation	Not significant
H16	IF → PE → FD	0.072 (1.7, 0.089)	0.019 (2.238, 0.025)	0.104 (2.505, 0.012)	18.30%	Full mediation	Significant

H10 proposed that IF mediates the relationship between GC and PC. The results show that the direct effect of GC on PC is significant (*β* = 0.112, *t* = 2.712, *p* = 0.007), and the indirect effect via IF is also substantial (*β* = 0.024, *t* = 2.379, *p* = 0.017). The total impact is significant (*β* = 0.136, *t* = 3.292, *p* = 0.001), with a VAF of 17.60%, indicating partial mediation. Therefore, H10 is supported.

H11 examined whether IF mediates the relationship between GC and PE. The direct effect of GC on PE is significant (*β* = 0.160, *t* = 4.122, *p* < 0.001), as is the indirect effect through IF (*β* = 0.022, *t* = 2.215, *p* = 0.027). The total impact remains significant (*β* = 0.181, *t* = 4.588, *p* < 0.001), with a VAF of 12.20%, suggesting partial mediation. Thus, H11 is supported.

H12 proposed that PC mediates the relationship between GC and FD. Although the total effect is significant (*β* = 0.095, *t* = 2.23, *p* = 0.026), neither the direct effect (*β* = 0.045, *t* = 1.07, *p* = 0.285) nor the indirect effect via PC (*β* = 0.011, *t* = 1.665, *p* = 0.096) reaches statistical significance. The VAF is 10.50%, indicating no mediation. Therefore, H12 is not supported.

H13 tested whether PE mediates the relationship between GC and FD. The direct effect of GC on FD is non-significant (*β* = 0.045, *t* = 1.07, *p* = 0.285), while the indirect effect via PE is significant (*β* = 0.023, *t* = 2.378, *p* = 0.017). The total impact is substantial (*β* = 0.095, *t* = 2.23, *p* = 0.026), and the VAF reaches 24.20%, indicating complete mediation. Hence, H13 is supported.

H14 examined IF as a mediator between GC and FD. The direct effect is non-significant (*β* = 0.045, *t* = 1.07, *p* = 0.285), and the indirect effect via IF is also non-significant (*β* = 0.012, *t* = 1.462, *p* = 0.144). Although the total effect is significant (*β* = 0.095, *t* = 2.23, *p* = 0.026), the VAF is only 12.60%, indicating no mediation. Thus, H14 is not supported.

H15 proposed that PC mediates the relationship between IF and FD. The direct effect is marginal but non-significant (*β* = 0.072, *t* = 1.70, *p* = 0.089), and the indirect effect via PC does not reach significance (*β* = 0.013, *t* = 1.707, *p* = 0.088). Despite a significant total effect (*β* = 0.104, *t* = 2.505, *p* = 0.012), the VAF is only 12.50%, indicating no mediation. Therefore, H15 is not supported.

H16 examined whether PE mediates the relationship between IF and FD. The indirect effect is significant (*β* = 0.019, *t* = 2.238, *p* = 0.025), while the direct effect remains non-significant (*β* = 0.072, *t* = 1.70, *p* = 0.089). The total effect is significant (*β* = 0.104, *t* = 2.505, *p* = 0.012), and the VAF reaches 18.30%, indicating full mediation. Accordingly, H16 is supported.

## 5 Discussion

### 5.1 Summary of main research findings

Synthesizing the above findings, the study’s structural model (Figure 1) demonstrates that GC elevates FD through the mediation of IF and PE. This influence relies entirely on the affective pathway (PE). The cognitive pathway (PC) exerts no significant effect. These results confirm that simulation games shape players’ fertility attitudes primarily through emotional engagement and affiliative connection, rather than through deliberate cognitive changes.

We further clarify the conceptualization of Fertility Desire (FD) in our model. For this study, FD is operationalized and interpreted primarily as an affective-preference dimension: the emotional and intuitive responses to childbearing and parenting scenarios (e.g., feelings of warmth, joy, and nurturance). This affective dimension differs from the broader, multi-component fertility decision-making process, which also includes deliberative calculations (e.g., economic costs) and social normative pressures. The GC → IF → PE → FD pathway (Figure 1) represents the core psychological mechanism through which virtual experiences influence and sustain an individual’s affective preference for fertility. Affective preference stems primarily from direct experience and implicit attitudes, driving rapid, intuitive reactions. In contrast, full fertility intention involves more deliberative processes, including the internalization of social norms. Thus, the findings of this study pertain specifically to affective-level fertility motivation. The observed associations operate through intermediary psychological processes, with emotional processes playing a prominent role.

GC exerts a significant positive direct effect on both PC and PE. This finding supports Horton and Wohl’s (1956) foundational theory, which holds that media users form one-way connections with virtual characters to elicit cognitive and emotional responses. The direct influence of GC on IF can be explained by Role Attachment Theory. Cohen (2018) posits that deep game immersion drives players to align their self-concept with that of the in-game character. Notably, GC has no direct effect on FD. This result indicates that the immediate impact of virtual experiences centers primarily on emotional satisfaction rather than long-term decision-making (Tukachinsky et al., 2020). The observed PE-PC link reveals that strong prosocial emotions (e.g., care and protection) in simulation games activate players’ affective heuristics. Under such influence, players tend to overlook the programmed nature of virtual characters and subconsciously attribute subjective realism to them. This cognitive shift further promotes the perception of these characters as sentient beings in need of nurturance.

Mediation analysis clarifies the specific pathways linking virtual experiences to real-world intentions. The analysis relies on the theoretically grounded Correlational Mediation Model, which aims to explore interconnections among variables rather than validate causal mechanisms. A key finding emerges: the positive effects of GC and IF on FD are fully mediated by PE, as confirmed by Hypotheses H13 and H16. This result strongly supports the central hypothesis that virtual characters function as Attachment Objects. It indicates that the emotional connections (PE) players form in the game can extend to real-world motivations. Thus, this emotional pathway serves as an essential Necessary Route (Konijn et al., 2007).

The significance of sequential paths (e.g., H10, H11) confirms that deep GC first enhances players’ IF with virtual characters (Pham et al., 2023). This identification strongly predicts deeper emotional involvement (Brown and Basil, 2010). The finding reflects a progressive psychological process: immersion fosters identification, identification triggers emotions, and emotions ultimately shape intentions. The cognitive path (Parasocial Cognition) shows no significant mediating role (H12, H15). This highlights that in high-emotional-load contexts such as simulation parenting (Gates, 2024), relationship development and decision-making motivation rely more on emotional connections than on purely rational cognitive frameworks.

Second, GC—defined as a psychological state of deep immersion—exerts multifaceted impacts. Players’ attention direction during immersion determines the primary mediating pathway for processing psychological motivations. Attention may focus on relationship-building (social orientation) (Rain and Mar, 2021), situational and motivational understanding (cognitive orientation) (Yu et al., 2023), or direct emotional resonance (emotional orientation) (Martin and Cohen, 2023).

Attention focus significantly influences cooperative decisions in games, and comprehensive information processing facilitates socially optimal choices (Lugrin et al., 2025). The life simulation games analyzed here integrate social interaction, narrative guidance, and emotional engagement. In these games, GC acts as a fundamental antecedent variable. It operates through multiple psychological processing pathways: promoting IF with virtual characters, deepening PC-based understanding of characters’ motivations, and enhancing PE. This mechanism confirms GC’s multifaceted effects, which initiate concurrent mediating pathways that collectively influence final FD. The model developed in this study thus provides nuanced insights into the intricate psychological processes linking virtual experiences to real-world intentions, strengthening theoretical explanatory power.

The observed pattern confirms that emotional and relational factors play a key role in the psychological pathway linking virtual experiences to real-world FD. Cognitive and rational analysis exerts a relatively limited impact. This finding highlights an intriguing conflict: though labeled “Simulation”—a term tied to cognitive engagement—these games primarily exert profound effects on players through emotional factors (e.g., empathy and attachment), rather than rational considerations of parenting responsibilities.

We propose an exploratory Emotional Compensation Hypothesis for the 18–35-year-old cohort—this study’s focus—who face real-world pressures. For this group, strong PE toward virtual characters within the game’s psychological safe zone may not translate directly into concrete fertility plans (Arima, 2025). Instead, these emotions act as a low-risk emotional resource, compensating for perceived deficits in real-life social support and emotional reserves (Weibel and Wissmath, 2024). The primary role of this virtual emotional fulfillment is to sustain, and even replenish, an individual’s positive imagination and emotional vitality.

This study uses cross-sectional data, which allows only correlational examination of the variables rather than definitive causal inferences. Thus, causal interpretations drawn from these findings require further validation via alternative research designs.

### 5.2 Theoretical and practical implications

#### 5.2.1 Theoretical contributions

First, this study challenges the claim that “Character Attachment Determines Real-World Behavior” (Zhang and Rau, 2023). In gaming contexts, emotional attachment to virtual characters can serve as an alternative source of emotional support, analogous to an incipient form of psychological safety. This interpretation warrants caution and further empirical validation across diverse contexts. Within this secure virtual environment, players with insecure attachment tendencies in real life can still develop positive, stable, and supportive PE toward virtual characters. These emotions—including being understood and supported by the virtual character (Salehi et al., 2023)—independently and positively predict FD. This challenges conventional attachment theory, which primarily emphasizes real-life constraints on relationships.

Second, this study advances understanding of in-game social relationships. It goes beyond the simplistic categorization of player-character relationships as merely IF (Mula-Márquez et al., 2024) and highlights the role of “Pseudo-Parenting” relationships in simulated parenting contexts. The model shows that the “Emotional Path” has a more substantial mediating effect than the “Cognitive Path.” This finding confirms that emotional connections (e.g., empathy and attachment) are paramount for translating virtual experiences into real-world motivations.

Third, this study tentatively explores an underexamined dimension of digital attachment research. It moves beyond the prevailing focus on romantic beliefs and mental health (Grosch, 2025) to investigate whether PSRS correlates with FD, a macro-demographic outcome. Our model exhibits modest explanatory power. The findings should be interpreted as a hypothetical framework rather than definitive causal evidence. This proposed connection aims to initiate an interdisciplinary dialogue about digital media’s subtle yet complex pathways that intersect with social reproductive motivations—pathways that merit deeper investigation.

In conclusion, this study identifies the core pathway: “immersive experience → IF/PSRS (emotion-driven) → FD.” This framework offers new insights into mechanisms shaping younger generations’ affective dispositions toward fertility in the digital age. Future public policy research should integrate digital media factors and examine their practical implications (Fauser et al., 2024).

#### 5.2.2 Practical implications

This study elucidates the psychological mechanism through which simulation games influence FD via an emotion-driven PSRS pathway. These findings provide empirical evidence and interdisciplinary insights to inform innovative digital media interventions in low-fertility societies.

We offer a novel perspective for advancing clinical and public health interventions. The research demonstrates that well-designed simulation games can act as low-cost Digital Psychological Tools (Gates, 2024). These games have two key potential applications. First, they can serve as Psychological Safe Bases, helping individuals with insecure attachment styles (e.g., anxious or avoidant attachment) practice emotional regulation and build connections in a secure virtual environment. This approach may indirectly improve their parenting intentions and self-efficacy (Camerman et al., 2023), providing a digital practice pathway to complement traditional attachment-based therapies. Second, these games serve as platforms for fertility cognition and emotional rehearsal, strengthening young adults’ understanding and positive attitudes toward family planning through empathy-integrated simulated narratives (Tukachinsky et al., 2020).

This study presents a design framework for the game industry to develop products with positive social benefits. The findings indicate that developers can intentionally foster positive player-virtual character connections by enhancing character authenticity, deepening emotional narratives, and designing supportive interactions (Feng, 2024). This framework encourages game designers to move beyond traditional entertainment and create Serious Games or educational simulations that promote psychological well-being. The approach integrates game design practices with social science objectives (Pacheco-Velázquez et al., 2023).

The study highlights the need to balance benefits and ethics in policy applications. Virtual experiences hold substantial intervention potential, but policymakers and developers must establish proactive ethical frameworks for their use in public health advocacy. This requires guarding against potential manipulation risks, avoiding the reinforcement of social stereotypes (Devillers and Cowie, 2023), and committing to bridging the digital divide to ensure fair and responsible interventions.

### 5.3 Limitations and future research directions

#### 5.3.1 Limitations

This study has three key limitations: theoretical application, methodology, and practical social applicability. First, the theoretical framework integrates Avatar Identification (GC), Role Attachment Theory, and PSRS, but relies disproportionately on Western attachment theory. This reliance overlooks cross-cultural variations in PE mechanisms. For example, IF in collectivist societies, individuals may prioritize group dynamics over individual emotions, potentially restricting the model’s generalizability (Rothbaum et al., 1982). Future research should incorporate a multidimensional fertility-intention scale to measure core variables comprehensively. Second, the methodology fails to account for demographic variables (e.g., marital status, education level, income), which limits the study’s comprehensiveness. Longitudinal or experimental designs will be required to infer causal relationships and capture the involved dynamic processes more effectively (Podsakoff et al., 2003). Finally, the model emphasizes the transfer of simulation game-derived skills. These skills may lack relevance to real-life contexts outside gaming—such as fertility decisions shaped by work pressures or family norms. This narrow focus limits the external validity of the findings, hindering their translation from laboratory settings to real-world contexts (Bagozzi and Van Loo, 1978).

#### 5.3.2 Future research directions

Future research can advance in three key areas: theoretical development, methodological enhancements, and cross-context application of findings. First, the theoretical model should be expanded to include cultural moderation variables. For example, integrating a collectivist perspective into the PE pathway can establish a cross-cultural framework. This framework examines how IF correlates with FD across different social norms (Van de Vijver and Leung, 2000). Second, longitudinal designs or experimental manipulations (e.g., random assignment to game types) should be adopted. Combining these with physiological measures—such as fMRI to index empathy activation—strengthens causal inference and reduces reliance on self-report data. A multidimensional FD scale enables more nuanced tests of the model’s differential predictive power across distinct fertility desire components, including cognitive appraisals and behavioral planning. Finally, the model can be applied to non-game virtual environments, such as social media or VR therapy. It also facilitates comparisons between different game types (e.g., competitive vs. simulation games) to explore PE transfer effects across broader population dynamics (Orben and Przybylski, 2019).

## 6 Conclusion

This study demonstrates that simulation games influence fertility desire through a sequential, affective pathway: GC → IF→PE. Based on this mechanism, we propose an Emotional Compensation Hypothesis. This hypothesis posits that in-game emotional experiences can act as a psychological resource to buffer fertility desire decline induced by real-world stressors. Our findings further indicate that simulation games may function as low-cost digital psychological tools. The study employs a cross-sectional design, limiting causal inference. Longitudinal research is thus required to verify these effects.

## Data Availability

The datasets presented in this study can be found in online repositories. The names of the repository/repositories and accession number(s) can be found in the article/supplementary material.
